# MALIGNANT MELANOMA PRESENTING AS MULTIPLE DISCHARGING SINUSES

**DOI:** 10.4103/0019-5154.62742

**Published:** 2010

**Authors:** Pradeep Vittal Bhagwat, Chandramohan Kudligi, Shashikumar BM, Arun Thirunavukkarasu, Mohan Eshwara Rao Shendre, Sujata Shivanand Giriyan

**Affiliations:** *From the Department of Skin and STD, Karnataka Institute of Medical Sciences, Hubli, Karnataka, India.*; 1 *From the Department of Skin and STD, Mandya Institute of Medical Sciences, Mandya, Karnataka, India.*; 2 *From the Department of Pathology, Karnataka Institute of Medical Sciences, Hubli, Karnataka, India.*

Sir,

Malignant melanoma is a malignant tumor arising from the epidermal melanocytes. It can be divided into three main subsets: the superficial spreading melanoma, the nodular melanoma and the lentigo maligna melanoma. Ulceration and bleeding from nodular melanomas occur frequently. About 5% of cutaneous melanomas present as an isolated, usually non-pigmented, subcutaneous or dermal nodule with no primary source of the tumor apparent.[1] Cutaneous malignancies may develop purulent discharge when there is secondary bacterial infection, especially in the ulcerative forms of the cancers. Cutaneous malignancies presenting as multiple discharging sinuses is rare. We report a case of malignant melanoma presenting as multiple discharging sinuses.

A 42-year-old male, resident of Hubli of Dharwad district, farmer by occupation, presented to us in February 2006 with history of swelling in the left groin of 15 days duration. It started as a small, painful nodule, rapidly increased in size and within 1 week, it became a severely painful, boggy swelling. The pain was very severe and patient was unable to walk or stand erect. Over a period of next 1 week, the swelling became fluctuant and there was discharge of foul smelling, blood stained, brownish discharge through multiple openings. The patient at this stage developed high grade fever associated with chills and rigors, malaise and fatigue. There was no history of genital ulcer, urethral discharge, or leg ulcer. There was no history of trauma at the site. There was no history of chronic cough or pain abdomen. There was history of weight loss of about 5 kg in the last couple of years. The patient had a history of multiple, heterosexual, unprotected exposure to a commercial sex worker 3 months back which was not followed by genital ulcer or urethral discharge. There was no history of similar complaints in the past. The patient had developed a blackish, nodular lesion over the left heel about 8 years back and it was removed by a surgeon. However, details of the diagnosis and treatment could not be obtained. The patient did not suffer from any other major medical or surgical illness in the past. The patient did not suffer from tuberculosis in the past. There was no family history of similar complaints. There was no family history of tuberculosis. The patient was not on any chronic medication. He was not an alcoholic or smoker. On examination, he was found to be ill-looking, febrile, moderately built, and nourished. Pulse, respiration, and blood pressure were within normal limits. There was no significant generalized lymphadenopathy. Systemic examination was unremarkable. Cutaneous examination revealed a solitary, tender, swelling measuring about 10 cm × 8 cm, situated over the upper one-third of the antero-medial aspect of the left thigh [Figure 1]. The swelling was fluctuant at the center, whereas it was firm to hard at the periphery. Skin over lesion was erythematous and edematous. Lesion was firmly attached to the underlying structures and was immovable. Few lymph nodes were palpable over the left inguinal region. They were about 4-5 cm in diameter, hard, non-tender, and immovable. Per-rectal examination was normal. There was a blackish, irregular, macular lesion, measuring 10 cm × 8 cm, situated over the left heel extending to the mid-foot [Figure 2]. There was no induration or tenderness over the lesion.

**Figure 1:**
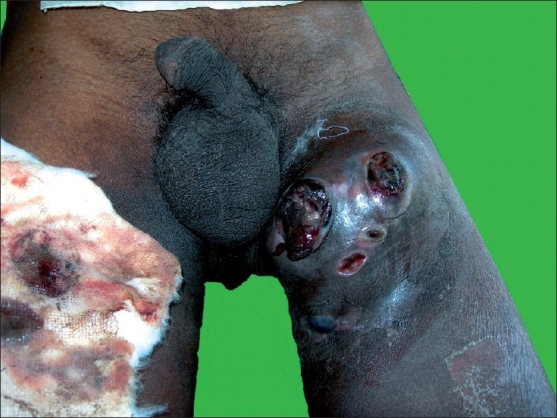
Boggy mass with multiple discharging sinuses over the antero-medial aspect of the left thigh

**Figure 2:**
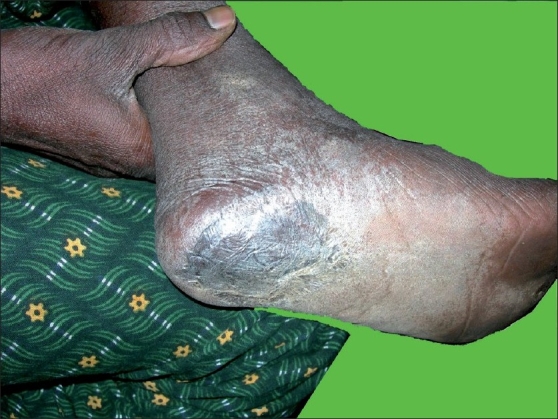
Recurrence of the malignant melanoma over the previously excised site over the left heel

Based on the history and clinical examination findings, we considered the differential diagnosis of lymphogranuloma venereum, cutaneous tuberculosis and actinomycosis. Routine hematological investigations, HIV antibodies, VDRL, radiological investigations, ultrasound examination of the abdomen, CT-scan of the chest, abdomen, pelvis and brain, CSF analysis, and montoux test were all within normal limits. Microscopic examination of the smear prepared from the pus revealed *Staphylococcus aureus*. The patient was treated with the syndromic approach of painful inguinal swelling. There was no improvement. FNAC was done from the enlarged left inguinal lymph nodes. It revealed fibrous papillary structures surrounded by tumor cells and lymphoid tissue with dispersed cell population arranged in loose aggregates and clusters with hemorrhagic and proteinaceous background. Tumor cells were round to oval in shape and had vesicular nuclei with prominent nucleoli and moderate amount of eosinophilic cytoplasm. There was clearing of chromatin. Nuclear pleomorphism was conspicuous. Atypical mitosis (ring mitosis), bizarre cells, intracytoplasmic melanin pigment, and intracytoplasmic vacuolation were well appreciated. Apart from tumor cells, inflammatory cells including lymphocytes, eosinophils and occasional mast cells, and lymphoglandular bodies were seen in the background. Extra-cellular brownish granular melanin pigment was also seen [Figure 3 and 4]. A biopsy was done from the periphery of the swelling, and histopathological examination confirmed the diagnosis of malignant melanoma [Figure 5 and 6]. Biopsy done from the blackish macule over the left heel also revealed features of malignant melanoma. It was concluded that the patient initially had malignant melanoma over the left heel which was inadequately excised and now presented with secondary deposits in the skin presenting as painful boggy swelling with multiple discharging sinuses and secondary deposits in the left inguinal region with the recurrence of the melanoma at the left heel. The patient was staged as TNM stage III b. The patient was referred to the department of surgery where the lesion was excised with 3 cm margin clearance and skin grafting was done. Complete groin dissection was done and complete therapeutic inguinal lymphadenectomy was done. Subsequently, the patient was given adjuvant radiotherapy.

**Figure 3:**
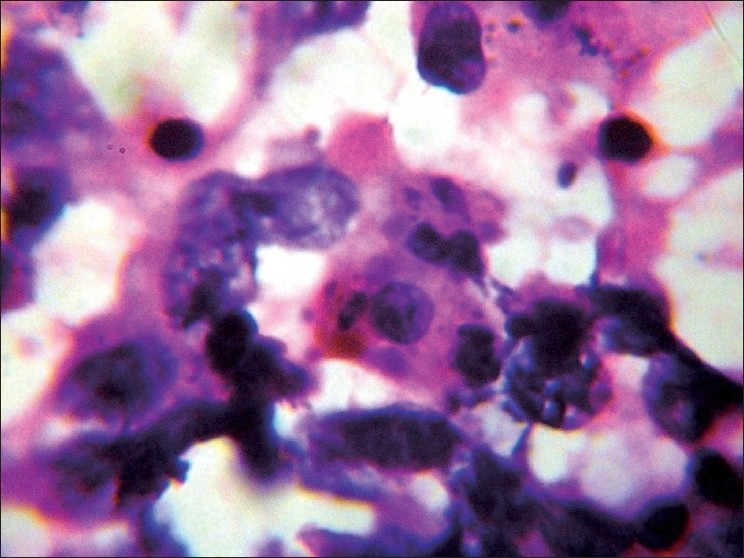
FNAC—tumor cell showing intracytoplasmic melanin pigment, intracytoplasmic vacuolation, atypical mitosis (ring mitosis), and bizarre cells. Low power

**Figure 4:**
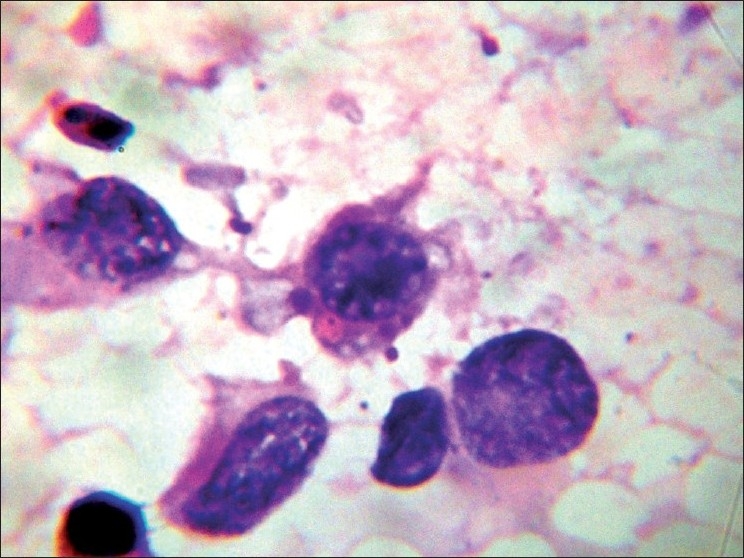
FNAC—tumor cell showing intracytoplasmic melanin pigment, intracytoplasmic vacuolation, atypical mitosis (ring mitosis), and bizarre cells. High power

**Figure 5:**
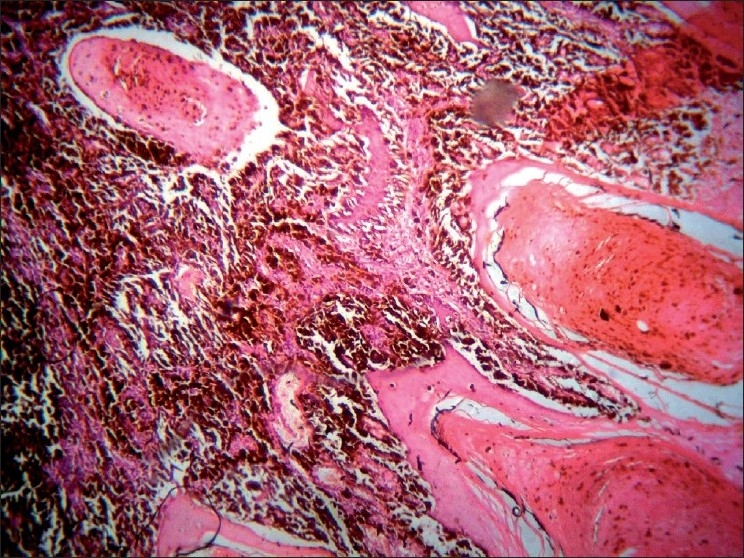
Histopathology showing features of malignant melanoma. Low power

**Figure 6:**
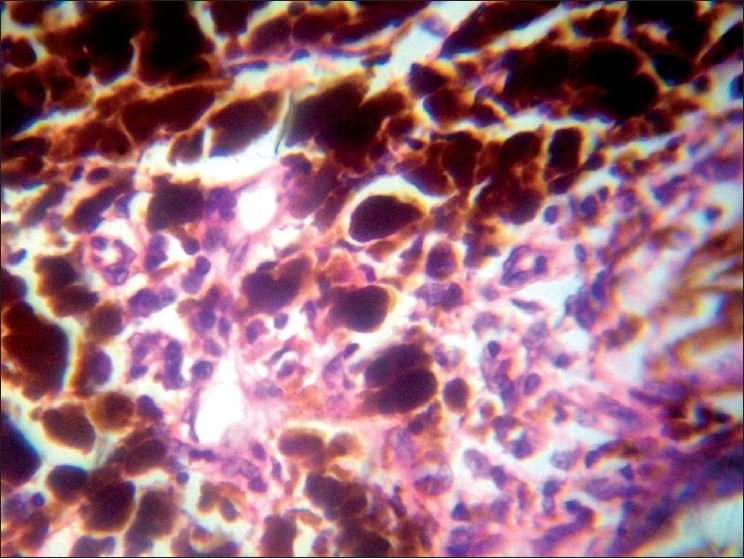
Histopathology showing features of malignant melanoma. High power

Apart from usual presentations, malignant melanoma has presented in many unusual ways like chronic non-healing ulcer,[2] pyogenic granuloma,[3] etc. Other malignancies like non-Hodgkins lymphoma[4] and histiocytosis-X[5] have presented as discharging sinuses. After extensive literature search, we could find two case reports where cutaneous malignancies presented as discharging sinuses. In the first case, a 28-year-old male presented with a swelling on the left side of the nose extending up to the left side of the forehead and encroaching the left side of the upper lip and angle of the mouth with a blackish, granular discharge from his left nostril and a couple of sinus openings on the left paranasal area. On detailed work-up, the case turned out to be sarcomatoid squamous cell carcinoma.[6] In yet another case report, a 62-year-old male patient presented with a long standing history of thyroid swelling which has metastasized to neck nodes and ulcerated over the midline resulting in a discharging sinus.[7] On detailed workup, this patient was found to have thyroid carcinoma. Our patient had malignant melanoma with secondary deposits in the skin and regional lymph nodes. The authors suggest that cutaneous malignancies may be kept as remote possibility in any unusual cases presenting as multiple discharging sinuses. The case is reported for its rarity of presentation.

## References

[1] MacKie RM (2004). Disorders of the cutaneous melanocyte. Rook's Textbook of Dermatology.

[2] Leight A (2006). A non-healing ulcerated fingertip following injury. J Fam Pract.

[3] Harrington P, O'kelly A, Trail IA, Freemont AJ (2002). Amelanotic subungual melanoma mimicking pyogenic granuloma in the hand. J Royal Coll Surg Edinburgh.

[4] Murtaza B, Khan NA, Nadeem A, Khan S, Saeed S (2007). Multiple perineal sinuses in non- hodgkin's lymphoma. J Coll Physicians Surg Pak.

[5] Sacks SH, Hall I, Ragge N, Pritchard J (1986). Chronic dermal sinuses as a manifestation of histiocytosis X. Br Med J (Clin Res Ed).

[6] Panda S (2009). A case of painful centrofacial nodule with discharging sinuses and blackish nasal discharge. Indian J Dermatol.

[7] Harish K (2008). Thyroid carcinoma with discharging sinus: A rarity: A case report. J Med Case Rep.

